# Toward Formal Analysis of Thermodynamic Stability: Le Chatelier—Brown Principle

**DOI:** 10.3390/e22101113

**Published:** 2020-10-01

**Authors:** Dmitry Gromov, Alexander Toikka

**Affiliations:** 1 Faculty of Applied Mathematics and Control Processes, St. Petersburg State University, 199034 Saint Petersburg, Russia; 2 Department of Chemical Thermodynamics and Kinetics, Institute of Chemistry, St. Petersburg State University, 199034 Saint Petersburg, Russia; a.toikka@spbu.ru

**Keywords:** equilibrium thermodynamics, stability condition, Gibbs stability condition, Le Chatelier— Brown principle, implicit function theorem, geometric thermodynamics, 80A10, 58C15, 26B10

## Abstract

In this contribution, we carry on with the research program initiated in *J. Math. Chem., 58(6), 2020*. Using the methods from geometric thermodynamics, we formally derive and analyze different conditions for thermodynamic stability and determine the limits of their use. In particular, we study, in detail, several versions of the Le Chatelier—Brown principle and demonstrate their application to the analysis of thermodynamic stability.

## 1. Introduction

The conditions of thermodynamic stability belong to the basic principles of general and chemical thermodynamics and are of the utmost importance for various fundamental and applied problems. Recall, for instance, the quotation from Herbert Callen in [1]: “Considerations of stability lead to some of the most interesting and significant predictions of thermodynamics”. Thus it is not strange that there are different variants of the stability conditions, both in classical monographs, e.g., [2,3,4] and also in research papers, e.g., [5,6,7,8,9,10,11]. The main difference between these formulations is the choice of the initial thermodynamic potentials, as well as the set of additional conditions imposed on the thermodynamic system. While most criteria are formulated in terms of the stability matrix, that is, the matrix formed by the second derivatives of thermodynamic potentials, there are several different approaches based upon expressing stability conditions in terms of inequalities involving partial derivatives of different thermodynamic potentials under different fixations of variables. Such approaches have a long history and date back to the works of Josiah Willard Gibbs [12] and Paul Ehrenfest [13]. In these papers, some initial formulations were proposed that were later extended and generalized. However, no formal mathematical analysis of these conditions and the limits of their applicability has been undertaken to date.

The idea of geometric interpretation of thermodynamics, originally suggested by Gibbs at the end of the 19th century, later formulated within the context of contact geometry by Robert Hermann in 1973 in [14], and further developed in a number of seminal works [15,16,17,18], forms a natural framework for a rigorous study of (equilibrium) thermodynamic systems. There have been a series of works extending this geometric approach to the study of dynamical evolution of thermodynamic systems, see, e.g., [19,20,21,22,23,24] and references therein. See also [25] for a comparison of different approaches.

In our research, we take a different route and apply the developed geometric approach to the rigorous mathematical analysis of stability conditions for an equilibrium thermodynamic system. In doing so, we further the research program initiated in [26]. Specifically, using the contact geometric formulation we describe the equilibrium energy manifold as an *n*-dimensional Legendre manifold of a particular thermodynamic differential 1-form, [27], and apply the tools from linear algebra and multivariable calculus, in particular the implicit function theorem and its ramifications, [28,29]. Along these lines, we not only formally derive some classical relations such as the Le Chatelier—Brown principle, but also carry out a rigorous mathematical analysis of these relations and determine the limits of their applicability. It is worth noting that the formal analysis of the studied relations require the use of rather non-trivial results from the matrix theory, some of which are not presented in the classical text books, e.g., [30], and hence were proved in the paper.

The paper is organized as follows: in Section 2, we introduce basic thermodynamic axioms and use them to derive some classical results from equilibrium thermodynamics. We also give a formal mathematical definition of generic thermodynamic stability and present initial results aimed at characterizing this property. In Section 3, the implicit function theorem is formulated and its application is illustrated. In particular, we give a previously obtained formulation of the Gibbs stability criterion and carry out a detailed analysis of possible situations that can occur when this criterion does not hold. Section 4 is devoted to the thorough analysis of the Le Chatelier—Brown principle. It is shown that while the respective inequalities can be used to indicate instability, they cannot guarantee that the system is stable. The paper is concluded with a discussion and two Appendices. Appendix A containing necessary results from the matrix analysis, while Appendix B contains proofs of some theorems that were removed from the main body of the paper to make it more straightforward.

### Notation

We use straight capital letters to denote matrices and vectors, slanted capital vectors for thermodynamic variables and functions and small letters for auxiliary parameters and also for specific values of extensive thermodynamic parameters, as defined in Section 2.2. The set of indices is denoted by *N*, N={1,…,n}. Let M be a matrix, we denote by M[i,j] the submatrix obtained by removing the *i*th row and the *j*th column of M, i,j∈N. If I⊂N and J⊂N are two subsets on the set of indices, we denote the submatrix obtained by removing respective rows and columns by M[I,J]. Conistently with the introduced notation, M[I,∅] and M[∅,J] denote the matrix M with only rows, resp. only columns removed. If i=j, the submatrix M[i,i] is called the *principal submatrix* of M and denoted by M[i]. Similarly, we define M[I] for I⊂N. The cardinality of a set *I* is denoted by |I|. Further, ${\mathrm{col}}_{i}M$ denotes the *i*th column of M. The set of vectors with strictly positive components is denoted by $R_{+}^{n}=\left\{{\left(x_{1},\ldots ,x_{n}\right)}^{⊤}\in R^{n}|x_{i}>0,\;i=1,\ldots ,n\right\}$.

## 2. Basic Ingredients from Equilibrium Thermodynamics

### 2.1. Internal Energy Function

We denote by $Y={\left[Y_{1},\;\ldots ,\;Y_{n+1}\right]}^{⊤}\in R_{+}^{n+1}$ the n+1-dimensional column vector of (strictly positive) extensive parameters, e.g., *S*, *V*, $m_{i}$ (entropy, volume, and quantities of substances, respectively) and define $Y_{0}=U\left(Y_{1},\ldots ,Y_{n+1}\right)$ to be a sufficiently smooth function that corresponds to the internal energy of the system. The intensive parameters $X_{i}$, which correspond to *T* (temperature), −p (pressure), $\mu_{i}$ (chemical potential of compound *i*), etc., are defined as $X_{i}=\frac{\partial U}{\partial Y_{i}}\left(Y_{1},\ldots ,Y_{n+1}\right)$.

The function *U* is postulated to be positively homogeneous of degree 1, i.e., we have

(1) $$U\left(\alpha Y_{1},\ldots ,\alpha Y_{n+1}\right)=\alpha U\left(Y_{1},\ldots ,Y_{n+1}\right),\;\alpha \in R_{+}.$$

By Euler’s homogeneous function theorem we have

(2) $$U\left(Y_{1},\ldots ,Y_{n+1}\right)=\sum_{i}\frac{\partial U(Y_{1},\ldots ,Y_{n+1})}{\partial Y_{i}}Y_{i}=\sum_{i}X_{i}Y_{i}.$$

Using rather straightforward manipulations, we recover the equation

(3) $$\sum_{i}\frac{\partial^{2}U\left(Y_{1},\ldots ,Y_{n+1}\right)}{\partial Y_{i}\partial Y_{j}}Y_{i}=\sum_{i}\frac{\partial X_{j}}{\partial Y_{i}}Y_{i}=\sum_{i}\frac{\partial X_{i}}{\partial Y_{j}}Y_{i}=0,\;j=1,\ldots ,n,$$

which is known as the *Gibbs–Duhem equation*. The first consequence of (3) is that the Hessian matrix of *U*, denoted by $D^{2}U$, is singular: $D^{2}U\cdot Y=0$. Finally, we will often make use of the *Maxwell reciprocal relations*$\frac{\partial X_{i}}{\partial Y_{j}}=\frac{\partial^{2}U}{\partial Y_{i}\partial Y_{j}}=\frac{\partial X_{j}}{\partial Y_{i}}$.

### 2.2. Stability Condition

Rather informally, we can say that a thermodynamic system is stable at some state $\bar{Y}$ if a sufficiently small perturbation applied to the system will be attenuated with time and will not lead to any change in the system state. We give a formal definition of local (generic) stability in terms of the Hessian of the internal energy. It should be noted that the proposed definition is formulated in terms of second-order partial derivatives and can be applied only when the respective matrix of second derivatives is well defined. Later on, we will consider the critical, i.e., boundary cases and point out possible approaches to analyzing them.

Beyond that, the suggested approach provides a characterization of stability only within a sufficiently small neighborhood of the point of interest. To infer the system behavior beyond that limit, higher order derivatives are to be analyzed. Keeping these reservations in mind, we proceed to the definition.

**Definition** **1.** *A thermodynamic system in energy-based representation is said to be*locally generically stable*(later on, simply stable) at $\bar{Y}=\left[{\bar{Y}}_{1},\;\ldots ,\;{\bar{Y}}_{n+1}\right]$ if the Hessian matrix $D^{2}U\left(\bar{Y}\right)$ is positive semi-definite with a single zero eigenvalue corresponding to the eigenvector ${\left[{\bar{Y}}_{1},\;\ldots ,\;{\bar{Y}}_{n+1}\right]}^{⊤}$.*

This specific structure can be characterized in a number of ways. Below, we present a result that will be used later on.

**Theorem** **1.** *Let* M *be an* [k×k] *positive semi-definite matrix. The following two conditions are equivalent:*

*1*. M *has a single zero eigenvalue and* $v={\left[\begin{array}{cccc} v_{1}, & v_{2}, & \ldots & v_{k} \end{array}\right]}^{⊤}\in R_{+}^{k}$ *is the corresponding right eigenvector;*
*2*. *For all indices* i∈{1,…,k} *, the principal submatrices* M[i] *are positive definite.*

**Proof.**  See Appendix B. □

Note that the property of the energy Hessian with one zero eigenvalue is a *structural invariant* as follows from the Gibbs–Duhem equations. It is therefore of interest to determine the points where a second zero eigenvalue appears as such points correspond to the true tipping points, where the system loses its stability.

Before we proceed with further stability analysis, we note that it is often more convenient to work with a different representation of the energy function. Such a representation can be obtained by fixing one variable, say, $Y_{n+1}$, and defining a *specific* energy function, i.e., the energy function

(4) $$u\left(y_{1},\ldots ,y_{n}\right)=\frac{1}{Y_{n+1}}U\left(Y_{1},\ldots ,Y_{n},\;Y_{n+1}\right)=U\left(y_{1},\ldots ,y_{n},\;1\right),$$

where the variables $y_{i}$ are the ratios $y_{i}=\frac{Y_{i}}{Y_{n+1}}$. The specific energy function is not homogeneous any longer, since

$$u\left(\alpha y_{1},\ldots ,\alpha y_{n}\right)=U\left(\alpha y_{1},\ldots ,\alpha y_{n},\;1\right)\neq \alpha U\left(y_{1},\ldots ,y_{n},\;1\right)=\alpha u\left(y_{1},\ldots ,y_{n}\right).$$

We can rewrite (4) to express the relation between $u(y_{1},\ldots ,y_{n})$ and $U(Y_{1},\ldots ,Y_{n+1})$ as

(5) $$Y_{n+1}u\left(y_{1},\ldots ,y_{n}\right)=U\left(Y_{1},\ldots ,Y_{n+1}\right).$$

Applying the chain rule to (5), one can write the relationship between the first-order derivatives of both energy functions:

$$\frac{\partial U}{\partial Y_{i}}=Y_{n+1}\sum_{k=1}^{n}\frac{\partial u}{\partial y_{k}}\frac{\partial y_{k}}{\partial Y_{i}},\;i=1,\ldots ,n.$$

Noting that

$$\frac{\partial y_{k}}{\partial Y_{i}}=\left\{\begin{array}{cc} 0, & i\neq k\\ \frac{1}{Y_{n+1}}, & i=k, \end{array}\right.$$

we get

(6) $$\frac{\partial U}{\partial Y_{i}}=Y_{n+1}\frac{\partial u}{\partial y_{i}}\frac{1}{Y_{n+1}}=\frac{\partial u}{\partial y_{i}},\;i=1,\ldots ,n.$$

This result agrees with the fact that the first-order derivative of a 1st order homogeneous function is a 0th order homogeneous function and hence, the first-order derivatives of both energy functions are equal.

Following the same logic, one can obtain the relationship between the second-order derivatives:

(7) $$\frac{\partial^{2}U}{\partial Y_{i}\partial Y_{j}}=\frac{1}{Y_{n}}\frac{\partial^{2}u}{\partial y_{i}\partial y_{j}},\;i,j=1,\ldots ,n-1.$$

This result implies that the Hessian of the specific energy function normalized w.r.t. the variable $Y_{i}$ is equal to the principal submatrix $D^{2}U\left[i\right]$ taken with the factor $Y_{i}$. Since $Y_{i}>0$, this implies the following result.

**Lemma** **1.** *Let $\bar{Y}\in R_{+}^{n}$ be a vector of extensive state variables. The following two statements are equivalent:*

*1*. *The energy functon $U(Y_{1},\ldots ,Y_{n})$ is stable at $\bar{Y}$;*
*2*. *The Hessians of specific energy function formulated with respect to the variables $y_{i}$ are positive definite at*
  $$\bar{y}=\left({\bar{Y}}_{1}/{\bar{Y}}_{i},\ldots ,{\bar{Y}}_{i-1}/{\bar{Y}}_{i},{\bar{Y}}_{i+1}/{\bar{Y}}_{i},\ldots ,{\bar{Y}}_{n+1}/{\bar{Y}}_{i}\right)$$
*for all i=1,…,n.*

The fundamental problem of equilibrium thermodynamics consists of determining the conditions under which the system passes from stability to instability. This corresponds to the situation when the energy Hessian has at least two zero eigenvalues. We recall that the first zero eigenvalue is structurally determined, and hence cannot vary. Therefore, the loss of stability corresponds to the appearance of a second zero eigenvalue. The following theorem gives the required characterization.

**Theorem** **2.** *Let M be an [k×k] positive semi-definite symmetric matrix. The following two conditions are equivalent:*

*1*. *M has a zero eigenvalue of algebraic multiplicity 2 and one of the right eigenvectors corresponding to the zero eigenvalue has all nonzero components.*
*2*. *For all indices i∈{1,…,k}, the principal submatrices M[i] are positive semi-definite of rank k−2.*

**Proof.**  See Appendix B. □

We will not consider the case when the transition from stability to instability corresponds to the simultaneous appearance of more than two zero eigenvalues, as such a situation is non-generic. To give an idea, we note that a space of all [3×3] real symmetric matrices has dimension 6 and the space of all such matrices with a double zero eigenvalue has dimension 3, while the set of [3×3] real symmetric matrices with 3 zero eigenvalues has dimension 0, as the only matrix satisfying this condition is the zero matrix.

Theorem 2 tells us that when studying the stability of the system with the energy function $U(Y_{1},\ldots ,Y_{n+1})$, it is sufficient to consider *any* order *n* principal submatrix of the energy Hessian. When a zero eigenvalue appears, we conclude that the system state is located on the stability border. Now, recall that any such submatrix enjoys the same properties as the Hessians of the specific energy function taken with respect to some extensive variable. Therefore, it appears to be convenient to work with a Hessian of the *specific* energy function. Let us assume, for certainty, that this function is obtained by fixing the last extensive variable $Y_{n+1}$. This assumption will be the starting point for our subsequent analysis.

From now on, we adopt the following convention: the specific energy function and the specific extensive variables are denoted by lower-case letters, while the intensive variables are still denoted by capital letters. That is to say, we will write $u(y_{1},\ldots ,y_{n})$ and $X_{i}=\partial u/\partial y_{i}$.

## 3. Application of the Implicit Function Theorem

### 3.1. Implicit Description of the Equilibrium Energy Manifold and the Implicit Function Theorem

The energy equilibrium manifold can be considered as an *n*-dimensional manifold embedded in the n+1-dimensional space and implicitly defined by

$$\Psi \left(y_{0},y_{1},\ldots ,y_{n}\right)=y_{0}-u\left(y_{1},\ldots ,y_{n}\right)=0.$$

Alternatively, one can embed this manifold into a higher-dimensional space of dimension 2n+1 with coordinates $\left(y_{0},y_{1},\ldots ,y_{n},X_{1},\ldots ,X_{n}\right)$, which we will refer to as the *thermodynamic state space*. Following this approach, the energy equilibrium manifold U is defined by the following system of equations:

(8) $$\left\{\begin{array}{c} \phi_{1}=y_{0}-u\left(y_{1},\ldots ,y_{n}\right)=0\\ \phi_{2}=X_{1}-\frac{\partial u}{\partial y_{1}}\left(y_{1},\ldots ,y_{n}\right)=0\\ \vdots \\ \phi_{n+1}=X_{n}-\frac{\partial u}{\partial y_{n}}\left(y_{1},\ldots ,y_{n}\right)=0. \end{array}\right.$$

We write (8) as $\Phi (y_{0},y,X)=0$, where $\Phi (y_{0},y,X)$ is a smooth vector-valued function, $\Phi :R^{2n+1}\to R^{n+1}$. Obviously, $\left(y_{1},\ldots ,y_{n}\right)$ form a set of independent variables, i.e., one can express $y_{0}$ and $X_{i}$, i=1,…,n in terms of $y_{j}$, j=1,…,n. However, other variables can be chosen to form a (local) coordinate system on U. These variables are characterized using the implicit function theorem.

Below, we will briefly outline the mathematical apparatus of implicit function theorem and its application to deriving thermodynamic quantities of interest. For more details, the interested reader is referred to the paper [26].

The Jacobian matrix of $\Phi (y_{0},y,X)$ writes as

(9) $$\begin{array}{ll} & \;\;\;\;y_{0}\;\;\;\;\;y_{1}\;\;\;\;\;\;y_{2}\;\;\;\;\;\;\ldots \;\;\;\;\;y_{n}\;\;\;\;\;X_{1}\;\;X_{2}\;\ldots \;X_{n}\\ D\Phi (y_{0},y,X)\;= & \left(\begin{array}{ccccccccc} 1 & -u_{1} & -u_{2} & \ldots & -u_{n} & 0 & 0 & \ldots & 0\\ 0 & -u_{11} & -u_{12} & \ldots & -u_{1n} & 1 & 0 & \ldots & 0\\ 0 & -u_{21} & -u_{22} & \ldots & -u_{2n} & 0 & 1 & \ldots & 0\\ \vdots & \vdots & \vdots & \ddots & \vdots & \vdots & \vdots & \ddots & \vdots \\ 0 & -u_{n1} & -u_{n2} & \ldots & -u_{nn} & 0 & 0 & \ldots & 1 \end{array}\right) \end{array},$$

where $u_{i}$ and $u_{ij}$ denote partial derivatives: $u_{i}=\frac{\partial u(y_{1},\ldots ,y_{n})}{\partial y_{i}}$ and $u_{ij}=\frac{\partial^{2}u\left(y_{1},\ldots ,y_{n}\right)}{\partial y_{i}\partial y_{j}}$. The outer upper row in (9) indicates the variables to which we differentiate.

Let the point $\Xi =({\bar{y}}_{0},{\bar{y}}_{1},\ldots ,{\bar{y}}_{n},{\bar{X}}_{1},\ldots ,{\bar{X}}_{n})$ belong to the equilibrium energy manifold U, i.e., it holds that Φ(Ξ)=0. The implicit function theorem states that, in a sufficiently small neighborhood of Ξ, one can express n+1 variables as functions of the remaining *n* variables if the matrix formed by the columns corresponding to these n+1 variables is non-singular at Ξ.

The introduced framework allows for the efficient computation of partial derivatives of thermodynamic variables with respect to other variables if such partials exist. Suppose that we have determined that the set of (n+1) variables *Z* can be expressed through a complementary set of *n* variables *W*, where *Z* and *W* form a disjoint partition of the set of all thermodynamic variables, both extensive and extensive, i.e., Z∩W=∅, $Z\cup W=\{y_{0},y_{1},\ldots ,y_{n},X_{1},\ldots ,X_{n}\}$. The partial derivative $\frac{\partial z_{i}}{\partial w_{j}}\left(W\right)$, where $z_{i}\in Z$ and $w_{j}\in W$, can be written as

(10) $$\frac{\partial z_{i}}{\partial w_{j}}\left(W\right)=-\frac{\det\left(\left[\begin{array}{ccccc} C_{z_{1}} & \ldots & C_{w_{j}} & \ldots & C_{z_{n+1}} \end{array}\right]\right)}{\det\left(\left[\begin{array}{ccccc} C_{z_{1}} & \ldots & C_{z_{i}} & \ldots & C_{z_{n+1}} \end{array}\right]\right)},$$

where $C_{z_{i}}={\mathrm{col}}_{z_{i}}D\Phi$ is a shorthand for the column of (9) indexed by the variable $z_{i}\in Z$. Thus, the matrices in the preceding expression are formed by aligning the respective columns.

Note that the choice of dependent and independent variables is crucial here. Sometimes, students dealing with thermodynamics get a feeling that “everything can be differentiated with respect to everything”. Obviously, this is not quite true, as the subsequent analysis will show. However, there is a large number of possible variants and the particular choice of variables can prove to be decisive in analyzing the properties of a thermodynamic system.

**Remark** **1.** *Note that if $y_{0}$ is considered to be a dependent variable, it does not alter the respective determinant, i.e.,*

$$\det\left(\left[\begin{array}{ccccc} C_{y_{0}} & C_{z_{2}}\ldots & C_{z_{i}} & \ldots & C_{z_{n+1}} \end{array}\right]\right)=\det\left(\left[\begin{array}{ccccc} C_{z_{2}} & \ldots & C_{z_{i}} & \ldots & C_{z_{n+1}} \end{array}\right]\right)$$

*Thus, we can drop the first equation in* (8) *and consider only n implicit expressions. The respective matrix is [2n×2n] instead of [2n+1×2n+1]:*

(11) $$\begin{array}{ll} & \;\;\;\;\;\;\;\;y_{1}\;\;\;\;\;\;\;y_{2}\;\;\;\;\;\ldots \;\;\;\;\;\;\;y_{n}\;\;\;X_{1}\;X_{2}\;\ldots \;X_{n}\\ D\Phi (y_{0},y,X)[1]\;= & \left(\begin{array}{cccccccc} -u_{11} & -u_{12} & \ldots & -u_{1n} & 1 & 0 & \ldots & 0\\ -u_{21} & -u_{22} & \ldots & -u_{2n} & 0 & 1 & \ldots & 0\\ \vdots & \vdots & \ddots & \vdots & \vdots & \vdots & \ddots & \vdots \\ -u_{n1} & -u_{n2} & \ldots & -u_{nn} & 0 & 0 & \ldots & 1 \end{array}\right) \end{array},$$

*where we write $D\Phi \left(y_{0},y,X\right)\left[1\right]$ to denote the principal minor, obtained by deleting the first row and the first column.*

### 3.2. Gibbs Stability Condition

In this subsection, we formally state the Gibbs stability criterion and expand upon its physical implications.

Consider a sequence of different sets of dependent and independent variables

(12) $$\begin{array}{ccc} Z_{1}=\left\{X_{1},\;X_{2},\;\ldots ,\;X_{n-1},\;X_{n}\right\} & \; & W_{1}=\left\{y_{1},\;y_{2}\;\ldots ,\;y_{n-1},\;y_{n}\right\}\\ Z_{2}=\left\{y_{1},\;X_{2},\;\ldots ,\;X_{n-1},\;X_{n}\right\} & \; & W_{2}=\left\{X_{1},\;y_{2},\;\ldots ,\;y_{n-1},\;y_{n}\right\}\\ Z_{3}=\left\{y_{1},\;y_{2},\;\ldots ,\;X_{n-1},\;X_{n}\right\} & \; & W_{3}=\left\{X_{1},\;X_{2},\;\ldots ,\;y_{n-1},\;y_{n}\right\}\\ \ldots & \; & \ldots \\ Z_{n}=\left\{y_{1},\;y_{2},\;\ldots ,\;y_{n-1},\;X_{n}\right\} & \; & W_{n}=\left\{X_{1},\;X_{2},\;\ldots ,\;X_{n-1},\;y_{n}\right\}, \end{array}$$

where we dropped $y_{0}$ in all sets of dependent variables, as suggested in Remark 1. Furthermore, let $D^{2}u_{1}^{k}$ denote the square matrix of second partial derivatives of *u* w.r.t. $\left(y_{1},\ldots ,y_{k}\right)$.

Now, the Gibbs stability criterion reads as follows (see [26] for the formal derivation and further detail).

**Theorem** **3.** *Let $\Xi =({\bar{y}}_{0},\bar{y},\bar{X})$ be a point on the equilibrium energy manifold U, such that $\det\left(D^{2}u_{1}^{i}\left(\Xi \right)\right)\neq 0$ for all i=1,…,n−1. Then, the determinant of the Hessian of u evaluated at* *Ξ is equal to the product of first-order partial derivatives*

(13) $$\det\left(D^{2}u\left(\Xi \right)\right)=\prod_{i=1}^{n}\frac{\partial X_{i}}{\partial y_{i}}\left(W_{i}\right){|}_{\Xi },$$

*where $W_{i}$ are defined in* (12).

The straightforward corollary of Theorem 3 is that the thermodynamic system is locally stable at Ξ if the product of the first derivatives of thermodynamic potentials obtained for different choices of dependent and independent parameters is non-zero (positive). However, since we are interested in determining the conditions under which the system is located on the stability boundary, we might wish to consider, in more detail, the case when the product (13) is equal to zero. In particular, we would like to know if it is possible to focus on considering a single product term instead of the whole chain.

We recall that the product (13) can be written in terms of determinants of respective matrices as

$$\begin{array}{c} \prod_{i=1}^{n}\frac{\partial X_{i}}{\partial y_{i}}\left(W_{i}\right)={\left(-1\right)}^{n}\frac{\det\left(\left[\begin{array}{cccc} C_{y_{1}} & C_{X_{2}} & \ldots & C_{X_{n}} \end{array}\right]\right)}{\det\left(\left[\begin{array}{cccc} C_{X_{1}} & C_{X_{2}} & \ldots & C_{X_{n}} \end{array}\right]\right)}\times \frac{\det\left(\left[\begin{array}{cccc} C_{y_{1}} & C_{y_{2}} & \ldots & C_{X_{n}} \end{array}\right]\right)}{\det\left(\left[\begin{array}{cccc} C_{y_{1}} & C_{X_{2}} & \ldots & C_{X_{n}} \end{array}\right]\right)}\times \\ \ldots \times \frac{\det\left(\left[\begin{array}{cccc} C_{y_{1}} & \ldots & C_{y_{n-1}} & C_{X_{n}} \end{array}\right]\right)}{\det\left(\left[\begin{array}{cccc} C_{y_{1}} & \ldots & C_{X_{n-1}} & C_{X_{n}} \end{array}\right]\right)}\times \frac{\det\left(\left[\begin{array}{cccc} C_{y_{1}} & \ldots & C_{y_{n-1}} & C_{y_{n}} \end{array}\right]\right)}{\det\left(\left[\begin{array}{cccc} C_{y_{1}} & \ldots & C_{y_{n-1}} & C_{X_{n}} \end{array}\right]\right)}. \end{array}$$

The numerator of the last term corresponds to the Hessian $D^{2}u$ and, hence, the last term turns equal to zero if there is a zero eigenvalue, that is the system is on the boundary of stability. On the other hand, the expressions in the denominators of the product terms correspond to the principal minors of the Hessian. If at least one minor equals zero, the whole expression turns out to be badly defined, as some of the fraction will take on an infinite value.

It is thus of interest to understand the conditions under which the above situation may occur. We will approach this issue from a geometrical viewpoint. As said above, we will consider the generic case when there is a single zero eigenvalue. Let v be the eigenvector corresponding to the zero eigenvalue. Following Theorem 1, we conclude that all principal submatrices of $D^{2}u\left(\Xi \right)$ are non-degenerate if, and only if, the vector v does not contain zero components (note that the sign of the respective components is not relevant here).

**Figure 1:**
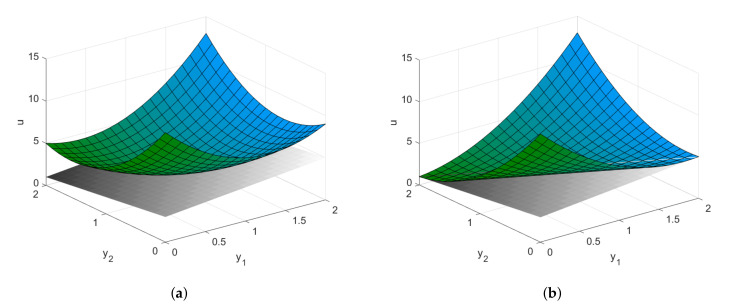
(**a**) Convex energy function; (**b**) The energy function gets flat in one direction. Two local approximations of a hypothetical energy function. The gray plane is the supporting hyperplane to the surface at the point of interest.

It is a known fact that the eigenvectors of the energy Hessian define the principal directions of the paraboloid (respectively, its level sets) that locally approximates the function $u(y_{1},\ldots ,y_{n})$ at Ξ. At a regular point, this paraboloid is non-degenerate elliptic, i.e., all its sections are either ellipses or ellipsoids. This paraboloid gets degenerate at the point where its Hessian has a zero eigenvalue. The eigenvector corresponding to the zero eigenvalue is particularly important as it defines the direction in which the paraboloid “gets flat”, that is, it becomes locally linear (see Figure 1 for an illustration of the difference). Whether this flat direction indicates an inflection point or just a local flattening depends on the higher order derivatives of *u* computed along this specific direction.

**Figure 2:**
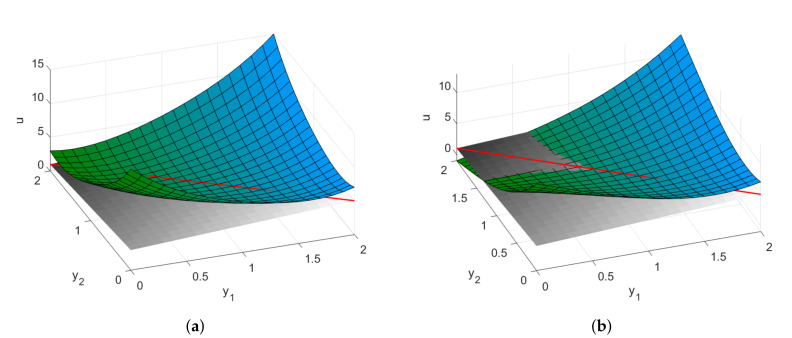
(**a**) A “valley”: locally flat convex function. This case is referred to as a “*neutral equilibrium*”; (**b**) An inflection point. The system is unstable. Two possible cases corresponding to a zero eigenvalue. The direction of the corresponding eigenvector is shown by the red line.

Note that in Figure 1 and in the subsequent figures, we draw a supporting hyperplane (see, e.g., [31] (Ch. 5) for a formal definition) to illustrate the convexity property of a surface or the lack thereof. Intuitively, a function is locally convex at the point Ξ if the graph of the function in a neighborhood U(Ξ) of this point is located on the side of the supporting hyperplane and touches the hyperplane at the single point Ξ. In this sense, Figure 1b depicts a semi-convex function that touches the supporting hyperplane along a line. Furthermore, Figure 2 illustrates the difference between two cases that occurs when the Hessian turns out to have a zero eigenvalue. In this case, the decision about the stability or instability of the system at point Ξ should be made upon further analysis of the higher order derivatives. Figure 2a illustrates the case of a flat, but still convex, function, while Figure 2b depicts a non-convex function, with an inflection point determined by the third-order derivatives.

To illustrate the scenario where there is at least one zero component of the eigenvector corresponding to the zero eigenvalue, we go back to the original representation of the energy function. Let $\bar{y}=\left({\bar{y}}_{1},\ldots ,{\bar{y}}_{n}\right)$ be a state such that there exists $v\in R^{n}$, satisfying $D^{2}u\left(\bar{y}\right)v=0$, i.e., v is the eigenvector corresponding to the zero eigenvalue. Furthermore, assume that the *i*th component of v is equal to zero. Then, the energy function *U* will be approximately flat for all points

$$y\left(\varepsilon \right)=\left({\bar{y}}_{1}+\varepsilon v_{1},\ldots ,{\bar{y}}_{i-1}+\varepsilon v_{i-1},{\bar{y}}_{i},{\bar{y}}_{i+1}+\varepsilon v_{i+1},\ldots ,{\bar{y}}_{n}+\varepsilon v_{n}\right),$$

where ε is a sufficiently small number. We can immediately conclude that degenerate principal submatrices can appear in the case where the direction in which the energy surfaces flattens is such that one or more variables have to be constant.

The preceding analysis indicates the following scenarios of the transition to instability. When the system state crosses the stability border, both the determinant $\det\left(D^{2}u\left(\Xi \right)\right)$ and the respective product $\prod_{i=1}^{n}\frac{\partial X_{i}}{\partial y_{i}}\left(W_{i}\right){|}_{\Xi }$ turn into zero, while the principal minors remain positive. As the system states moves beyond the stability border, the determinant turns negative and the principal minors subsequently become equal to zero. In this case, the expression $\prod_{i=1}^{n}\frac{\partial X_{i}}{\partial y_{i}}\left(W_{i}\right){|}_{\Xi }$ is not well defined any longer. Alternatively, when the system is on the border of stability, both the determinant $\det\left(D^{2}u\left(\Xi \right)\right)$ and some of the principal minors $\det\left(D^{2}u\left(\Xi \right)\left[i\right]\right)$ become equal to 0. In this case, the product of the partial derivatives, as shown above, may not be well defined. We note that the latter situation is very specific and occurs only under the condition that the eigenvector, corresponding to the zero eigenvalue, has at least one zero element. Such a condition is not generic as it can be destroyed by an arbitrary perturbation of the system parameters. However, it may be of importance in the subsequent analysis of the higher order derivatives of the energy function.

Finally, note that the transition to instability is always accompanied by zeroing of the determinant, $\det\left(D^{2}u\left(\Xi \right)\right)=0$, as follows from the Cauchy interlacing theorem: if any principal minor of a symmetric positive semi-definite matrix M is equal to zero, then the matrix M has a zero eigenvalue.

## 4. Le Chatelier–Brown Principle

### 4.1. A Classical Formulation of the Le Chatelier—Brown Principle

In this section, we consider a different concept, which is, however, somewhat closely related to the notion of thermodynamic stability. Namely, the Le Chatelier—Brown principle says that “*when a system at equilibrium experiences external impacts, the latter cause counter-action forces to appear in the system that tend to decrease the effect of these impacts*”. This formulation resembles the definition of stability used in the theory of dynamical systems: “*when disturbed from a condition of equilibrium, the system develops forces or moments that restore the original condition*”. The Le Chatelier—Brown principle remains valid for all regular states where the energy function has a strict minimum. However, it may not hold any longer if the energy manifold is not convex. This forms a connection to the results presented above.

We start by considering the variant of the Le Chatelier—Brown principle proposed by Paul Ehrenfest in 1909. It is formulated as an inequality expressed in terms of first-order partial derivatives of the same variables, but with a different choice of dependent variables:

(14) $${\left(\frac{\partial y_{i}}{\partial X_{i}}\right)}_{X_{k}}\geq {\left(\frac{\partial y_{i}}{\partial X_{i}}\right)}_{y_{k}},$$

where ${\left(\frac{\partial y_{i}}{\partial X_{i}}\right)}_{y_{k}}=\frac{\partial y_{i}}{\partial X_{i}}\left(y_{1},\;\ldots ,y_{k-1},\;y_{k+1},\;\ldots ,y_{n},\;X_{k}\right)$. Using the expression (10), we can compute the respective partial derivatives

$${\left(\frac{\partial y_{i}}{\partial X_{i}}\right)}_{X_{k}}=\frac{\det\left(D^{2}u\left[i\right]\right)}{\det\left(D^{2}u\right)}\;\mathrm{and}\;{\left(\frac{\partial y_{i}}{\partial X_{i}}\right)}_{y_{k}}=\frac{1}{u_{ii}}$$

Note that the first partial derivative exists only under the condition that the Hessian $D^{2}u$ is non-singular, as otherwise the denominator would turn to zero. Using the Jacobi identity (A1), we get

$$\frac{\det\left(D^{2}u\left[i\right]\right)}{\det\left(D^{2}u\right)}={\left[{\left(D^{2}u\right)}^{-1}\right]}_{ii},$$

that is, this version of the Le Chatelier—Brown principle boils down to saying that the *i*th diagonal element of the energy Hessian matrix is larger or equal to the inverse of the respective diagonal element of the original Hessian:

(15) $${\left[{\left(D^{2}u\right)}^{-1}\right]}_{ii}\geq \frac{1}{u_{ii}}.$$

At this point, Theorem A3 can be used to show that the inequalities (15), resp. (14) hold for all i=1,…,n if the energy Hessian is positive definite, i.e., the system is stable. Recall that for the positive semi-definite Hessian $D^{2}u$, the expression (14) is not defined any longer, as the first partial derivative may turn to infinity.

Note that the result, converse to that stated in Theorem A3, is not true even under certain additional conditions such as, e.g., the positiveness of the diagonal elements of the matrix M. Thus, one cannot conclude that $D^{2}u$ is positive definite if (14) holds for all *i*. However, this condition can be used to reject specific cases. Namely, we can conclude the $D^{2}u$ is not positive definite if (14) does not hold for at least one i∈{1,…,n}.

### 4.2. An Extended Version of the Le Chatelier—Brown Principle

We have considered the formulation of the Le Chatelier—Brown principle in which we compared the cases where the set of independent variables consisted of all *X*’s and of all *Y*’s with a single $X_{i}$. This setup can be extended by allowing *Y*-variables to substitutte the respective *X*-variables one by one. The respective sets of dependent ($Z_{i}$) and independent ($W_{i}$) variables would be as follows:

(16) $$\begin{array}{ccc} Z_{0}=\left\{y_{1},\;y_{2},\;\ldots ,\;\;y_{n}\right\} & \; & W_{0}=\left\{X_{1},\;X_{2}\;\ldots ,\;X_{n}\right\}\\ Z_{1}=\left\{X_{1},\;y_{2},\;\ldots ,\;\;y_{n}\right\} & \; & W_{1}=\left\{y_{1},\;X_{2}\;\ldots ,\;X_{n}\right\}\\ Z_{2}=\left\{X_{1},\;X_{2},\;y_{3},\;\ldots ,\;y_{n}\right\} & \; & W_{2}=\left\{y_{1},\;y_{2},\;X_{3}\;\ldots ,\;X_{n}\right\}\\ \ldots & \; & \ldots \\ Z_{i-1}=\left\{X_{1},\;\ldots ,\;X_{i-1},\;y_{i},\;\ldots ,\;y_{n}\right\} & \; & W_{i-1}=\left\{y_{1},\;\ldots ,\;y_{i-1}\;X_{i},\;X_{i+1},\;\ldots ,\;X_{n}\right\}\\ Z_{i}=\left\{X_{1},\;\ldots ,\;X_{i-1},\;X_{i+1},\;y_{i},\;y_{i+2},\;\ldots ,\;y_{n}\right\} & \; & W_{i}=\left\{y_{1},\;\ldots ,\;y_{i-1},\;y_{i+1},\;X_{i},\;X_{i+2},\;\ldots ,\;X_{n}\right\}\\ \ldots & \; & \ldots \\ Z_{n}=\left\{X_{1},\;\ldots ,\;X_{i-1},\;X_{i+1}\;\ldots ,\;X_{n},\;y_{i}\right\} & \; & W_{n}=\left\{y_{1},\;\ldots ,\;y_{i-1},\;y_{i+1},\;\ldots ,\;y_{n},\;X_{i}\right\}. \end{array}$$

Therefore, we can write down the partial derivatives $\frac{\partial y_{i}}{\partial X_{i}}$ for different sets of dependent and independent variables (16) as follows:

$$\begin{array}{cc} \frac{\partial y_{i}}{\partial X_{i}}\left(W_{0}\right)=\frac{\det\left(D^{2}u\left[i\right]\right)}{\det\left(D^{2}u\right)} & \;\frac{\partial y_{i}}{\partial X_{i}}\left(W_{1}\right)=\frac{\det\left(D^{2}u\left[1,i\right]\right)}{\det\left(D^{2}u\left[1\right]\right)}\\ \ldots & \ldots \\ \frac{\partial y_{i}}{\partial X_{i}}\left(W_{i-1}\right)=\frac{\det\left(D^{2}u\left[\left\{1,\ldots ,i\right\}\right]\right)}{\det\left(D^{2}u\left[\left\{1,\ldots ,i-1\right\}\right]\right)} & \;\frac{\partial y_{i}}{\partial X_{i}}\left(W_{i}\right)=\frac{\det\left(D^{2}u\left[\left\{1,\ldots ,i+1\right\}\right]\right)}{\det\left(D^{2}u\left[\left\{1,\ldots ,i-1,i+1\right\}\right]\right)}\\ \ldots & \ldots \\ \frac{\partial y_{i}}{\partial X_{i}}\left(W_{n}\right)=\frac{1}{u_{ii}} \end{array}$$

Now, we can formulate an extended version of the Le Chatelier–Brown theorem that was initially formulated in [32] and [33] (as well as in some other form, using second derivatives of thermodynamic potentials in [7,9]), albeit in a less formal way.

**Theorem** **4.** *If the system is stable at $\Xi =({\bar{y}}_{0},\bar{y},\bar{X})$ then for any i=1,…,n the following chain of inequalities holds at* Ξ *and for the sets $W_{i}$ defined in* (16):

$$\frac{\partial y_{i}}{\partial X_{i}}\left(W_{0}\right){|}_{\Xi }\geq \frac{\partial y_{i}}{\partial X_{i}}\left(W_{1}\right){|}_{\Xi }\geq \ldots \geq \frac{\partial y_{i}}{\partial X_{i}}\left(W_{n}\right){|}_{\Xi }.$$

**Proof.**  The proof is based upon the observation that any inequality can be written as

$$\frac{\det\left(D^{2}u\left[I\cup j\right]\right)}{\det\left(D^{2}u\left[I\right]\right)}{|}_{\Xi }\geq \frac{\det\left(D^{2}u\left[I\cup j\cup k\right]\right)}{\det\left(D^{2}u\left[I\cup k\right]\right)}{|}_{\Xi }$$

for some choice if indices *I*. This inequality holds as follows from Theorem A4. □

We first note that the formulation of the Le Chatelier—Brown principle as formulated in [13] (equation (14)) follows from Theorem 4. Similarly to the mentioned condition, Theorem 4 does not provide a sufficient condition for the stability of a system. However, similarly to the condition (14), Theorem 4 can be used to determine the cases where the system loses stability.

## 5. Discussion

In this paper, we presented further developments of the research program aimed at carrying out a rigorous mathematical analysis of classical relations and obtaining novel thermodynamic conditions in a systematic way. In particular, we analyzed the classical Gibbs stability condition and indicated several possible scenarios leading to the violation of these conditions, both accompanied and unaccompanied by a transition to instability. The obtained results form a basis for the analysis of high-order stability conditions that will be presented in the subsequent papers. We also formally derived several versions of the Le Chatelier—Brown principle and indicated the conditions under which these conditions can be used to conclude about the stability of a thermodynamic system. All presented results are based on the use of certain rather non-trivial results from matrix analysis that are formulated and proved in the paper. It is worth noting that the simplest cases following from the resulting equations are quite expected and conversant, e.g., it follows from Theorem 4 that the rate of changes in extensive properties increases with a decrease in the number of fixed intensive parameters. The discussion of some other more complicated cases would be a subject of our following works.

The presented results can be extended and generalized along several directions. One possible extension would consist in considering an irreversible version of the Le Chatelier–Brown principle following the line of research that was initiated in the works by I. Gyarmati [34] and later developed by M. Grmela and collaborators, see, e.g., [35]. Another possibility consists of making a bridge from the developed thermodynamic stability criteria to the second-law-based proof of Lyapunov stability in homogeneous nonequilibrium thermodynamic systems, see, e.g., [36]. These directions will be the subject of our future research.

## Appendix A. Necessary Results from Matrix Analysis

In this paper, we will mostly use classical results from matrix analysis. The interested reader can refer to [30] for more details. Below, we will present some results that are either not available from classical textbooks or require some additional elaboration.

**Theorem** **A1.**
*Cf. [37], Example 2.2] For a symmetric positive semi-definite matrix M, rank of any its principal submatrix M[I], I⊂N={1,…,n}, is equal to the rank of the matrix obtained by removing only the ith rows or only the ith columns, i∈I, of matrix M:*

rankM[I]=rankM[I,∅]=rankM[∅,I].

**Proof.** Let B be a principal submatrix of M, i.e., B=M[I]. Simultaneously permuting the rows and columns of M, we can represent M as

$$M=\left[\begin{array}{cc} B & C\\ C^{⊤} & D \end{array}\right].$$

Assume that $\mathrm{rank}\left(\left[\begin{array}{cc} B & C \end{array}\right]\right)>\mathrm{rank}B$ and choose the vectors x and y of appropriate dimensions such that $x^{⊤}B=0$, $x^{⊤}C\neq 0$, and $x^{⊤}Cy\neq 0$ (this is always possible because the matrix $\left[\begin{array}{cc} B & C \end{array}\right]$ has less linearly dependent rows than B). Now, we can write the respective quadratic form

$$\left[\begin{array}{cc} x^{⊤} & y^{⊤} \end{array}\right]\left[\begin{array}{cc} B & C\\ C^{⊤} & D \end{array}\right]\left[\begin{array}{c} x\\ y \end{array}\right]=2x^{⊤}Cy+y^{⊤}Dy,$$

where $y^{⊤}Dy\geq 0$ (note that D is a principal submatrix of M w.r.t. the indices N∖I). For any fixed y, one can choose x such that the sum $2x^{⊤}Cy+y^{⊤}Dy$ turns negative. This leads to a contradiction, hence we conclude that

$$\mathrm{rank}\left(\left[\begin{array}{cc} B & C \end{array}\right]\right)=\mathrm{rank}\left({\left[\begin{array}{cc} B & C \end{array}\right]}^{⊤}\right)=\mathrm{rank}\left(\left[\begin{array}{c} B\\ C^{⊤} \end{array}\right]\right)=\mathrm{rank}B.$$

□

There is a particularly useful relationship between the minors of a matrix and its inverse, that is referred to as the Jacobi identity in [30], albeit without a proof. Here, we prove a special version of it that we will need when deriving results on the Le Chatelier—Brown principle.

**Theorem** **A2.**
*Let $M\in R^{n\times n}$ be a non-singular matrix and let I⊂N={1,…,n} and $I^{c}=N\setminus I$ be a set of indices and its complement. Then the following identity holds:*

(A1) $$\det M^{-1}\left[I^{c}\right]=\frac{\det M[I]}{\det M}.$$

**Proof.** Let |I|=k. By simultaneously permuting the rows and columns we transform the matrix M into the matrix A such that A[{n−k+1,…,n}]=M[I]. With notation K={n−k+1,…,n} and $K^{c}=\left\{1,\ldots ,n-k\right\}$, this writes as M[I]=A[K]. Respectively, $A\left[K^{c}\right]=M\left[I^{c}\right]$ and the same for inverse matrices. Now, consider the matrix product

$$A\left(\begin{array}{cc} I_{k} & A^{-1}\left[K,K^{c}\right]\\ 0 & A^{-1}\left[K^{c}\right] \end{array}\right)=\left(\begin{array}{cc} A[K] & A[K,K^{c}]\\ A[K^{c},K] & A[K^{c}] \end{array}\right)\left(\begin{array}{cc} I_{k} & A^{-1}\left[K,K^{c}\right]\\ 0 & A^{-1}\left[K^{c}\right] \end{array}\right)=\left(\begin{array}{cc} A[K] & 0\\ A[K^{c},K] & I_{n-k} \end{array}\right)$$

and take the determinant of the first and the last expressions. This yields

$$\det A\det A^{-1}\left[K^{c}\right]=\det A\left[K\right],$$

whence—upon necessary substitutions— it follows that (A1). □

**Theorem** **A3.**
*Let $M\in R^{n\times n}$ be a symmetric positive-definite matrix. For any i=1,…,n, it holds that*

(A2) $${\left[M^{-1}\right]}_{ii}\geq \frac{1}{m_{ii}},$$

*that is, diagonal elements of the inverse matrix are not smaller than the inverse diagonal elements of the original matrix.*

**Proof.** Since M is symmetric and positive definite, it can be written as

$$M=P^{⊤}\Sigma P=B^{⊤}B,$$

where $B=\Sigma^{\frac{1}{2}}P$. Respectively, we have $M^{-1}=P^{⊤}\Sigma^{-1}P=B^{-1}B^{-⊤}$, where $B^{-⊤}={\left(B^{-1}\right)}^{⊤}$. Note that the *i*th diagonal element of M can be obtained as

$$m_{ii}=e_{i}^{⊤}Me_{i}=e_{i}^{⊤}B^{⊤}Be_{i}={∥Be_{i}∥}^{2},$$

where $e_{i}$ are the canonical Euclidean basis vectors. A similar expression can be written for ${\left[M^{-1}\right]}_{ii}$. Using the Cauchy–Bunyakovsky–Schwarz inequality, we get

$$m_{ii}{\left[M^{-1}\right]}_{ii}=∥Be_{i}{∥}^{2}{∥B^{-⊤}e_{i}∥}^{2}\geq {\langle Be_{i},B^{-⊤}e_{i}\rangle }^{2}={\langle e_{i},B^{⊤}B^{-⊤}e_{i}\rangle }^{2}={\langle e_{i},e_{i}\rangle }^{2}=1,$$

whence follows (A2). □

The following theorem presents an extension of the Jacobi identity. However, the authors are not aware whether it was mentioned previously.

**Theorem** **A4.**
*Let $M\in R^{n\times n}$ be a symmetric positive definite matrix. Then, it holds for any i,j∈{1,…,n}, i≠j that*

(A3) $$\frac{\det M[i]}{\det M}\geq \frac{\det M[\{i,j\}]}{\det M[j]}.$$

**Proof.** Since M is positive definite, all its minors are positive as well. Thus, we can rewrite (A3) as

(A4) $$\frac{\det M[i]}{\det M}\frac{\det M[j]}{\det M}\geq \frac{\det M[\{i,j\}]}{\det M}.$$

Using (A1) we get $\frac{\det M[\{i,j\}]}{\det M}=\det\left[M^{-1}\right]\left[{\left\{i,j\right\}}^{c}\right]={\left[M^{-1}\right]}_{ii}{\left[M^{-1}\right]}_{jj}-{\left[M^{-1}\right]}_{ij}{\left[M^{-1}\right]}_{ji}$, which can be rewritten as

(A5) $$\frac{\det M[\{i,j\}]}{\det M}=\frac{\det M[i]}{\det M}\frac{\det M[j]}{\det M}-{\left[M^{-1}\right]}_{ij}{\left[M^{-1}\right]}_{ji}.$$

Noting that the inverse to a symmetric positive definite matrix is a symmetric positive definite matrix as well, we conclude that ${\left[M^{-1}\right]}_{ij}={\left[M^{-1}\right]}_{ji}$ and hence, ${\left[M^{-1}\right]}_{ij}{\left[M^{-1}\right]}_{ji}={\left[M^{-1}\right]}_{ij}^{2}>0$. Substituting (A5) into (A4), we get the required result. □

## Appendix B. Proofs of the Theorems

**Proof of** **Theorem 1.** We first prove [1.]⇒[2.] Any principal submatrix of a positive semi-definite matrix is positive semi-definite. Thus, we need to show that all the principal submatrices are full rank. Since the components of the right eigenvector *v* are strictly positive (and hence, non-zero), no combination of k−1 columns of matrix M is linearly dependent. This implies that any submatrix formed by k−1 columns of M has full rank. According to Theorem A1, the respective principal sumbatrices, M[i], i∈{1,…,k}, have full rank as well. Therefore, all principal submatrices M[i] are positive definite as well as all their principal submatrices. Now consider [2.]⇒[1.] A positive-definite matrix has strictly positive eigenvalues, and thus is of full rank. If for some i∈{1,…,k}, M[i] has only positive eigenvalues and M is positive semi-definite, then, by the Cauchy interlacing theorem [30], M has a single zero eigenvalue. Let us assume that the eigenvector corresponding to this eigenvalue has a zero component at the *j*th position. In this case, the rank of the matrix, obtained by deleting the *j*th column from M is still equal to k−1 and hence, by Theorem A1, rank(M[j])≤k−1, which contradicts the assumption. □

**Proof of** **Theorem 2.** The proof of this theorem follows the same lines as in the proofs of previous theorem and hence will only be sketched. If a positive-definite symmetric matrix has two zero eigenvalues, then by Cauchy’s interlacing theorem, each of its principal submatrices will have at least 1 zero eigenvalue. Assume that there exists i∈{1,…,k} such that the principal submatrix M[i] has 2 zero eigenvalues. By Theorem A1, it holds that rank(M[i])=rank(M[∅,i])=k−3, thus there are two vectors $c^{\prime },c^{\prime \prime }\in R^{k-1}$ such that $M\left[\emptyset ,i\right]c^{\prime }=M\left[\emptyset ,i\right]c^{\prime \prime }=0$. Padding $c^{\prime }$ and $c^{\prime \prime }$ with 0 at *i*th position, we obtain two eigenvectors corresponding to a zero eigenvalue. However, both have at least one zero element, which contradicts the assumption. □
